# 2019 Annual Meeting of the Swiss Society for Sleep Research, Sleep Medicine, and Chronobiology (SSSSC)

**DOI:** 10.3390/clockssleep1040033

**Published:** 2019-09-20

**Authors:** Martin Hatzinger

**Affiliations:** SSSSC President, Psychiatric Services, Solothurner Spitäler AG, Solothurn, and Medical Faculty, University of Basel, Weissensteinstrasse 102, CH-4503 Solothurn, Switzerland; martin.hatzinger@spital.so.ch

We are pleased to present you with this Special Issue of *Clocks & Sleep*, the abstracts of the 2019 annual meeting of the Swiss Society for Sleep Research, Sleep Medicine, and Chronobiology (SSSSC), which took place in Fribourg, 27 and 28 June.

The 2019 meeting in Fribourg provided evidence-based education to advance the science and clinical practice of sleep medicine and sleep physiology, disseminated cutting-edge sleep and circadian research, promoted the translation of basic science into clinical practice, and fostered the future of the field by providing young clinicians and researchers with the opportunity to present their findings in talks and through posters, the abstracts of which can be found in this Special Issue.

Renowned international and national speakers provided comprehensive updates, educational workshops, and insights into novel scientific topics covering various areas of sleep research and sleep medicine. Special emphasis was given to the field of insomnia, sleep and cognition, sleep and development, oscillatory mechanisms, sleep apnea, and autoimmune disorders.

A data-flash and poster session allowed for the presentation of the latest results of current research. Most importantly, the meeting provided ample opportunities to bring together people to engage in discussions and plan clinical cases and scientific projects.

Taken together, we had an exciting, inspiring, and collaborative meeting in Fribourg!

## Abstract Content



**Session “Basic Science”**

**No.**

**Title**

**Author**

**Page**
1Waking up during the night—timing of microarousals in relation to the infraslow oscillation of mouse NREM sleepRomain Cardis4162Standing Sentinel during Human Sleep: Continued Evaluation of Environmental Stimuli in the Absence of ConsciousnessChristine Blume4163Basal forebrain contributions to brain state regulation during auditory learning and sleepArndt-Lukas Klaassen4174Optimising auditory stimulation of slow-wave sleep and associated memory benefitHolly Kings4175Locus coeruleus noradrenergic modulation of primary dorsal thalamus tunes the infraslow 0.02 Hz-oscillation of mouse NREM sleepAlejandro Osorio-Forero4186Prediction of sleep apnea severity using a portable electrocardiogram monitoring deviceMartin Brutsche4197Overnight changes in EEG slow wave slopes in the course of developmentValeria Jaramillo4198New learning during sleepSimon Ruch4209Diurnal changes in cortical glutamate + glutamine levels from childhood to adulthood and their relationship to sleepCarina Volk42010Evening caffeine intake induces alertness in teenagers, but does not affect melatonin levelsCarolin Reichert42111Changes in cross-frequency coupling following closed-loop auditory stimulation during NREM sleepElena Krugliakova42212Fear learning, slow-wave sleep, and trait anxiety: a conditioning studyImogen Birch42313Increased neuronal signatures of targeted memory reactivation during slow-wave up-statesEva Van Poppel42314Daily caffeine consumption reduces human grey matter in hippocampus independent of sleep pressureYu-Shiuan Lin42415The effects of repeated daytime caffeine intake and its cessation on nighttime sleepJanine Weibel42516The influence of pre-sleep social media consumption and relaxation on sleep qualitySelina Ladina Combertaldi42617Phase-coupled sleep oscillations within and between human hippocampus and neocortexRoy Cox426
**Session “Clinical Science”**
18REM-sleep related heart rate variability distinguishes between insomnia and normal sleepMarcia De Witte42719Sleep determinants of incident cardiovascular events: a prospective population-based studyCamila Hirotsu42820Circadian modulation of core body temperature in patients with central hypersomnia disordersFranziska Ryser42821Behavioural and regional EEG features of parasomnia episodes in disorders of arousalJacinthe Cataldi42922Nocturnal gamma-hydroxybutyrate (GHB) reduces cortisol awakening response and morning tryptophan catabolites in healthy volunteersDario Dornbierer43023Feeling awake during sleep: a high-density EEG assessment of sleep perception in good sleepers and patients with paradoxical insomniaSandro Lecci43124“Sleep well, my dear, to be fit for loving motherhood” – insomnia but not objective sleep deficits correlate with poor bondingThorsten Mikoteit43125The borderland between wakefulness and sleepDavid Schreier43226Hypoxic load and subjective daytime sleepiness in the HypnoLaus cohortMathieu Berger43327Epic dreaming disorder: clinical, polysomnographic, and dream features in 12 patientsCharlotte Krämer433


## Session “Basic Science”

### ***1. Waking Up during the Night—Timing of Microarousals in Relation to the Infraslow Oscillation of Mouse NREM Sleep*** 

CardisR.^1^LecciS.^2^FernandezL.^1^CherradN.^1^Osorio-ForeroA.^1^LüthiA.^1^1Department of Fundamental Neurosciences, UNIL, 1005 Lausanne, Switzerland2Centre d’investigation et de recherche sur le sommeil, CHUV, 1005 Lausanne, Switzerland

**Background:** Uninterrupted periods of sleep and wakefulness are an integral part of a healthy life cycle. However, brief awakenings also occur while being asleep. These are thought to appear randomly and are commonly considered as disruptive or even pathological elements of sleep. Furthermore, strategies to anticipate and prevent awakenings are currently missing. Here, we analysed the timing of microarousals in mouse non-rapid eye movement sleep (NREMS) in the light of a novel infraslow oscillation that segregates NREMS into periods of fragility and continuity.

**Methods:** Using polysomnographic recordings, we determined infraslow dynamics of sigma power (10–15 Hz) and microarousals. We used 6 h of total sleep deprivation to increase homeostatic sleep pressure, and 6 h- of selective REM sleep (REMS) deprivation using closed-loop feedback techniques to increase REMS pressure.

**Results:** We find that microarousals in mouse NREMS occur overwhelmingly time-locked during the fragility periods. When homeostatic sleep pressure was increased, fewer fragility periods were interrupted by microarousals. When only REMS pressure was augmented instead, fragility periods became primarily interrupted by transitions into REMS. Specifically, NREMS showed entries into REMS every ~50 or 100 s, thus coinciding with the periodicity of the infraslow oscillation. Instead, the frequency of microarousals was diminished.

**Conclusions:** We conclude that the timing of microarousals is gated by the fragility periods of the infraslow oscillation that constitute permissive windows for transitions out of NREMS, which can be allocated to NREMS–wake or NREMS–REMS transitions according to instantaneous pressures. These data provide a framework for the possibility of moment-to-moment assessments of sleep’s fragility to anticipate, and potentially protect, ill-timed awakenings.

**Funding:** No funding.

### ***2. Standing Sentinel during Human Sleep: Continued Evaluation of Environmental Stimuli in the Absence of Consciousness*** 

BlumeC.^1^,^2^,^3^,^4^del GiudiceR.^3^,^4^WislowskaM.^3^,^4^HeibD. P. J.^3^,^4^SchabusM.^3^,^4^1Centre for Chronobiology, Psychiatric Hospital of the University of Basel, 4002 Basel, Switzerland2University of Basel, Transfaculty Research Platform Molecular and Cognitive Neurosciences (MCN), 4002 Basel, Switzerland3University of Salzburg, Department of Psychology, Laboratory for Sleep, Cognition and Consciousness Research, 5020 Salzburg, Austria4University of Salzburg, Centre for Cognitive Neuroscience (CCNS), 5020 Salzburg, Austria

**Background:** Previously, stimuli such as a subject’s own name (SON) or a familiar voice have been shown to be processed preferentially during wakefulness. SONs and negative prosody have been shown to continue being salient even during unconscious non-rapid eye movement (NREM) sleep stage N2. Here, we investigated stimulus processing during wakefulness and across all the sleep stages (i.e., NREM sleep stages N1, N2, N3, and REM sleep). Furthermore, we also investigated oscillatory sleep electroencephalography (EEG) phenomena, i.e., sleep spindles and slow oscillations (SOs), and how their occurrence and characteristics relate to stimulus processing.

**Methods:** The modulation of stimulus processing by stimulus salience was studied by means of evoked oscillatory responses (event-related de/synchronisation [ERS/ERD]) and event-related potentials (ERPs) using high-density electroencephalography (EEG). Specifically, we varied stimulus salience on two dimensions: (i) subjective relevance and (ii) paralinguistic aspects of emotional relevance. To this end, we presented participants with SONs versus unfamiliar names (UNs) and additionally varied the familiarity of the voice, i.e., familiar versus unfamiliar voice (FV versus UFV).

**Results:** Analyses of ERS/ERD and ERPs reveal that the evaluation of voice familiarity continues during all of the NREM sleep stages, including N3 sleep and even REM sleep. Especially, UFV stimuli elicit larger responses in a 1 to 15-Hz range as well as larger K-complex-like responses, suggesting they continue being salient. Beyond this and contrasting earlier findings, we find that sleep spindles and the negative slope of SOs do not uniformly inhibit information processing, but inhibition seems to be scaled to stimulus salience.

**Conclusions:** The findings add to existing evidence for a “sentinel processing mode” of the human brain during sleep. This mode is characterised by the continued processing of environmental stimuli that may be followed by either an inhibitory sleep-protective response or awakening depending on the result of stimulus evaluation. Beyond this, it appears that even “transient oscillatory activity”, i.e., sleep spindles and slow oscillations, is sensitive to stimulus salience.

**Funding:** Konrad-Adenauer-Stiftung e.V. & Austrian Science Fund (FWF: W-1223, Y-777, J-4243)

### ***3. Basal Forebrain Contributions to Brain State Regulation during Auditory Learning and Sleep*** 

KlaassenA.-L.^1^,^2^HarveyM.^1^RaschB.^2^RainerG.^1^1Department of Medicine, University of Fribourg, CH-1700 Fribourg, Switzerland2Department of Psychology, University of Fribourg, CH-1700 Fribourg, Switzerland

**Background:** The basal forebrain (BF) projections play an important role in modulating neural network states, for example by enhancing cortical responsivity as well as contributing to wake/sleep regulation. The BF projections regulate cerebral cortical function by providing the major source of cholinergic as well as GABAergic and glutamatergic input to the neocortex.

**Methods:** Here, we aim to investigate the neuromodulatory influence of the BF on brain state regulation by combining optogenetic stimulation in the ventral pallidum of the BF with electrophysiology in the anterior cingulate cortex (ACC) in rats. To study the functional contribution of the BF projection system on learning behaviour, we developed an auditory discrimination paradigm. Rats learned to discriminate segments of different Fourier amplitude-matched classical music pieces using an operant conditioning paradigm.

**Results:** By optic BF activation (ChR2 general virus), our preliminary analyses revealed an increase in movement speed and impaired discrimination performance. By contrast, BF inhibition (Arch general virus) did not affect discrimination performance, but triggered a reduction in operant lever pressing. We also conducted sleep experiments, during which we manipulated the amount of non-rapid eye movement (NREM) sleep by BF optogenetics. We observed disturbed cortical slow wave activity associated with an increase in wakefulness following BF activation, whereas BF inhibition led to prolonged NREM sleep with elevated cortical delta oscillations.

**Conclusions:** Our findings shed new light on the role of the basal forebrain nucleus ventral pallidum in terms of regulating behaviour state, learning, and sleep.

**Funding:** Swiss National Science Foundation Doc.CH 168602.

### ***4. Optimising Auditory Stimulation of Slow-Wave Sleep and Associated Memory Benefits*** 

KingsH.^1^NavarreteM.^1^BergamoD.^1^SchneiderJ.^1^,^2^PereiraS.^1^CassonA.^2^LewisP.^1^1School of Psychology, Cardiff University, Cardiff, CF24 4HQ, Wales, UK2School of Electrical and Electronic Engineering, The University of Manchester, Manchester, M1 3BB, England, UK

**Background:** Closed loop auditory stimulation (CLAS) has repeatedly been shown to improve performance on a word pair association task. However, there is little evidence of whether this stimulation has similar effects on other memory tasks. We studied the effect of CLAS on two memory tasks: mnemonic similarity (MST) and serial reaction time (SRTT). Furthermore, we aimed to optimise CLAS by understanding the effect of varying the stimulation protocol upon ongoing oscillatory brain activity during S3 sleep. As such, stimulus duration was either 50 ms or 100 ms of pink noise. Meanwhile, the length of the inter stimulus interval (ISI) was varied between one, two and three slow oscillations.

**Methods:** Seventeen volunteers attended the lab for two nights, seven days apart. One night consisted of CLAS (STIM), the other consisted of no stimulation (SHAM). Stimulation was delivered during stage 2 and stage3 sleep.

**Results:** Pattern separation scores in the MST became significantly worse overnight in both conditions. In STIM there was a smaller and less significant reduction in performance overnight, however, there was no significant difference between conditions. There was also no clear trend when stimuli were divided based upon their difficulty. Recognition memory showed similar results, however, the hardest stimuli to distinguish were significantly less poorly affected in STIM than SHAM. Interestingly, STIM showed a non-significant trend to being detrimental to the easiest stimuli to distinguish compared to SHAM. Overnight changes in SRTT were not significant between conditions, but a qualitatively larger increase in speed occurred in STIM. Reaction times in a psychomotor vigilance task decreased overnight in STIM but increased in SHAM; however, the difference was not significant. There was a non-significant trend for larger ERP in STIM nights when stimuli of 100 ms were used. There appeared to be little difference in ERP when ISI was varied.

**Conclusions:** Our results provide trend-level support for the idea that CLAS enhances performance on these tasks; however, more data is needed to establish whether this is significant. Electrophysiology results warrant further analysis to understand the wider impact on sleep.

**Funding:** This project was funded as part of a BBSRC PhD studentship.

### ***5. Locus Coeruleus Noradrenergic Modulation of Primary Dorsal Thalamus Tunes the Infraslow 0.02-Hz Oscillation of Mouse NREM Sleep*** 

Osorio-ForeroAlejandroCardisRomainFernandezLauraDevenogesChristianeHornungJean-PierreLüthiAnitaDepartment of Fundamental Neurosciences, University of Lausanne, Lausanne 1005, Switzerland

**Background:** Non-rapid eye movement sleep (NREMS) of mouse and human shows an infraslow-oscillation (ISO, ~50-s period) in the spindle power band (sigma, 10–15 Hz) that generates alternating states of high and low arousability, which is phase-locked to variations in heart rate and pupil diameter. The neural circuits underlying the ISO are currently unknown. A candidate nucleus is the locus coeruleus (LC), which is key for arousal and attention during wakefulness, but that remains active during NREMS and controls sleep spindle generation and sensory arousability.

**Methods:** We studied projections from noradrenergic LC neurons to the dorsal and reticular thalamic nuclei through the retrograde viral labeling of dopamine-ß-hydroxylase (DBH)-expressing neurons in 7 to 10-week-old C57Bl/6J mice. To assess their role in NREMS, we inhibited α-adregenic and ß-adrenergic receptors through the local infusion of atenolol (5 mM) and prazosin hydrochloride (0.1 mM) into primary thalamic somatosensory areas, and recorded pre-injection and post-injection sleep–wake behaviour from ZT1-7 in freely moving animals implanted with ipsilateral somatosensory (S1) local field potential electrodes together with polysomnography (*n* = 5).

**Results:** Anatomical projections from LC to the thalamus arise from densely packed subgroups of DBH-expressing neurons in the anterior half of the LC. Compared to the baseline, the pharmacological inhibition of thalamic adrenergic receptors reduced the power of the oscillatory sigma pattern in NREMS in the ~50-s range (from 0.12 ± 0.06 nV2 to 0.04 ± 0.02 nV2) and suppressed the phase relation of discrete spindles to the ISO (r of Rayleigh from 0.57 ± 0.05 to 0.30 ± 0.06). Moreover, the absolute sigma activity in S1 during the phases of low sigma power of ISO was increased (from 0.31 ± 0.08 a.u. to 0.42 ± 0.12 a.u.).

**Conclusions:** We identify the anatomical and functional relation between the LC and the reticular and dorsal thalamus during NREMS, and propose that the phasic release of noradrenaline into thalamic sleep spindle-generating circuits as part of the circuitry underlying the mammalian ISO.

**Funding:** This project is funded by an international PhD Fellowship of UNIL-FBM and by SNF.

### ***6. Prediction of Sleep Apnea Severity Using A Portable Electrocardiogram Monitoring Device*** 

BatyF.^1^BoeschM.^1^WidmerS.^1^AnnaheimS.^2^FontanaP.^2^CamenzindM.^2^RossiR.^2^SchochO.^1^BrutscheM.^1^1Lung Center, Cantonal Hospital St. Gallen, 9007 St. Gallen, Switzerland2Laboratory for Biomimetic Membranes and Textiles, Empa, 9014 St. Gallen, Switzerland

**Background:** Sleep apnea syndrome (SAS) is diagnosed and graded by polysomnography (PSG) based on the number of apnea–hypopnea events per hour of sleep (apnea–hypopnea index, AHI). PSG is expensive and technically complex; therefore, simpler and more cost-effective devices for screening and follow-up are desirable. In this context, a wearable electrocardiogram (ECG) acquisition system was developed by the Swiss Federal Laboratories for Materials Science and Technology (Empa). The aim of the present study was to evaluate the value of overnight ECG recordings to assess apnea severity using an ECG belt in a population of patients with suspicion of SAS.

**Methods:** The ECG belt consists of a semi-elastic polyester backbone with directly embroidered Ag/Ti-coated PET yarn forming the electrodes. Patients with suspicion of SAS were recruited. An ECG signal was recorded in every patient using both the belt and PSG. Heart rate variability (HRV) analysis was carried out, and a prediction model using support vector machine was applied. The prediction accuracy of apnea severity was assessed for both signal acquisition systems.

**Results:** Whole night measurements were performed on 241 patients. The median AHI was 21 [IQR: 7–40]/h and 65% of the patients suffered from SAS (AHI >10/h). The ECG belt had an accuracy, sensitivity, and specificity of 72%, 70%, and 74%, respectively, to discriminate individuals without SAS and individuals with SAS of different degrees of severity, which was comparable to PSG. The prediction accuracy was comparable to PSG and particularly suitable for the discrimination between patients with no/mild SAS versus moderate/severe SAS.

**Conclusions:** In individuals suspected of SAS, a novel textile-based ECG belt was able to detect HRV-based disturbances of the vegetative nervous system, which allowed for the assessment of sleep apnea severity. The follow-up of patients with SAS could be complemented by HRV, giving additional insights into the cardiovascular stress level during sleep.

**Funding:** The study was supported by an unconditional research grant by the Lungenliga St. Gallen—Appenzell and an institutional grant by the Kantonsspital St. Gallen.

### ***7. Overnight Changes in EEG Slow Wave Slopes in the Course of Development*** 

JaramilloV.^1^,^2^VolkC.^1^,^2^FurrerM.^1^,^2^FattingerS.^1^,^2^KurthS.^3^LustenbergerC.^4^HuberR.^1^,^2^,^5^1Children’s Research Center, University Children’s Hospital Zurich, 8032 Zurich, Switzerland2Child Development Center, University Children’s Hospital Zurich, 8032 Zurich, Switzerland3Baby Sleep Lab, University Hospital Zurich, 8032 Zurich, Switzerland4Institute of Human Movement Sciences and Sport, ETH Zurich, 8032 Zurich, Switzerland5Department of Child and Adolescent Psychiatry and Psychotherapy, Psychiatric Hospital University of Zurich, 8032 Zurich, Switzerland

**Background:** The restorative function of sleep has been linked to an overall reduction in synaptic strength. The synaptic homeostasis hypothesis proposes that synaptic renormalization during sleep occurs in a “smart” manner: while most synapses are selected for renormalization, some synapses are protected from down-selection. During brain development, major synaptic restructuring occurs, and impairments in synaptic down-selection during sleep may be associated with cognitive and memory deficits in clinical populations. Synaptic down-selection has been connected to slow waves during non-rapid eye movement (NREM) sleep. While both the amplitude and the slope of slow waves decrease during sleep, the slope is thought to most directly reflect synaptic strength, when controlling for amplitude changes across the night. Here, we aimed to examine overnight slope changes in the course of development in a region-dependent and amplitude-dependent manner.

**Methods:** All-night high-density electroencephalographam (EEG) was recorded in 60 healthy subjects aged between 8–30 years (mean ± s.e.m., 18.9 ± 0.8 years, 25 females). To control for amplitude changes across the night, we matched slow waves from the first and the last hour of NREM sleep according to their amplitude.

**Results:** Our results show that slow wave slopes decreased from the first to the last hour of NREM sleep (F_time_ (1,58) = 289.6, *p* < 0.001) across the cortex. This global overnight slope reduction was largest in children and decreased with age (*r* = −0.510, *p* < 0.001). Topographical analyses revealed regional differences in the slow wave slope decrease, which were dependent on amplitude. Specifically, for small amplitude waves, the decrease was smallest over an occipital area, whereas for large amplitude waves, the decrease was smallest over a central area (F_occipital_ (7,392) = 4.81, *p* < 0.001, F_central_ (7,392) = 8.75, *p* < 0.001).

**Conclusions:** The larger slope decrease in children suggests a boosted down-selection of synapses during sleep in childhood, which in turn might be related to increased plasticity during brain maturation. Regional differences in the extent of slow wave slope reduction may reflect the “smart” down-selection process or, alternatively, indicate amplitude dependent differences in the generation of slow waves. These results reveal the need to consider age when using the overnight slope change as a biomarker in clinical populations.

**Funding:** SNF Sinergia CRSII3_160803, CRPP “Sleep and Health”, SNF 320030_153387

### ***8. New Learning during Sleep*** 

RuchSimon^1^,^2^ZüstMarc Alain^1^,^2^WiestRoland^3^HenkeKatharina^1^,^2^1Institute of Psychology, University of Bern, Switzerland2Center for Cognition, Learning and Memory, University of Bern, Switzerland3Support Center for Advanced Neuroimaging (SCAN) and Institute of Diagnostic and Interventional Neuroradiology, University Hospital of Bern, Switzerland

### ***9. Diurnal Changes in Cortical Glutamate + Glutamine Levels from Childhood to Adulthood and their Relationship to Sleep*** 

VolkC.^1^,^2^JaramilloV.^1^,^2^StudlerM.^1^,^2^FurrerM.^1^,^2^O’Gorman TuuraR.^1^,^3^HuberR.^1^,^2^,^4^1Children’s Research Center, University Children’s Hospital Zurich, 8032 Zurich, Switzerland2Child Development Center, University Children’s Hospital Zurich, 8032 Zurich, Switzerland3MR Center, University Children’s Hospital Zurich, 8032 Zurich, Switzerland4Department of Child and Adolescent Psychiatry and Psychotherapy, Psychiatric Hospital University of Zurich, 8032 Zurich, Switzerland

**Background:** Sleep slow waves play a crucial role in maintaining cortical plasticity, which is a process that is especially important in the developing brain. Children show a considerably larger overnight decrease in slow-wave activity (SWA; EEG power between 1–4.5 Hz), which constitutes the primary EEG marker for the restorative function of sleep. In healthy young adults, we recently demonstrated that this EEG marker correlates with the overnight reduction in cortical glutamate + glutamine (GLX) levels assessed by magnetic resonance spectroscopy (MRS), suggesting GLX as a promising biomarker for the interplay between cortical plasticity and SWA. Since brain maturation depicts a time window of increased cortical plasticity and increased sleep need, we examined age-related overnight changes in GLX and SWA from childhood to adulthood.

**Methods:** The MRS spectra of 46 subjects (mean ± s.e.m., 16.5 ± 0.78 years, 24 females) were measured in the left parietal lobe in the evening before a night of sleep and in the subsequent morning using a 3T GE MR 750 scanner. GLX concentrations were quantified using a locus coeruleus (LC) model and corrected for a partial volume contamination of cerebrospinal fluid (CSF). The decrease of SWA in the course of the night was calculated as a percentage reduction from the sleep cycle with maximal SWA to the last sleep cycle.

**Results:** The overnight changes in GLX correlated negatively with age (*p* = 0.01, *r* = −0.4), differed between children (<12 years) and adults (>18 years; *p* < 0.05; pairwise comparisons using a Mann–Whitney U-Test, fdr adjusted) and changed its sign from positive to negative (mean children = 6.3%, mean adults = −5.4%)). No other metabolites showed a significant correlation for overnight changes with age (all *p* > 0.2). We found no correlation between changes in GLX and (a) the reduction in SWA (*p* = 0.4), (b) the duration of wake after sleep onset, or (*p* = 0.4) (c) the duration of REM sleep (*p* = 0.6).

**Conclusions:** Given that children and adolescents show a higher decline in SWA over the course of the night, we expected that GLX reductions would be more pronounced within this cohort. Here, we find the contrary: namely, a more pronounced overnight decrease in adults compared to children. If reorganizational processes during brain development might mask diurnal changes in GLX levels or if these manifest after cortical maturation is completed needs to be elucidated in the future.

**Funding:** Swiss National Science Foundation (320030_153387) and the Clinical Research Priority Program (CRPP) Sleep and Health of the University of Zurich.

### ***10. Evening Caffeine Intake Induces Alertness in Teenagers, but Does Not Affect Melatonin Levels*** 

ReichertC.^1^,^2^VeitzS.^1^,^2^LinY.-S.^1^,^2^,^3^WeibelJ.^1^,^2^GarbazzaC.^1^,^2^MeyerM.^1^,^2^,^4^SlawikH.^1^,^2^,^4^CajochenC.^1^,^2^1Centre for Chronobiology, Psychiatric Hospital of the University of Basel, 4002 Basel, Switzerland2Transfaculty Research Platform Molecular and Cognitive Neurosciences, University of Basel, 4002 Basel, Switzerland3Neuropsychiatry and Brain Imaging, Psychiatric Hospital of the University of Basel, 4002 Basel, Switzerland4Clinical Sleep Laboratory, Psychiatric Hospital of the University of Basel, 4002 Basel, Switzerland

**Background:** Adolescents suffer from short sleep duration and frequently consume caffeine in the evening. Observational studies indicate worse academic performance and lower quality of sleep in teenagers reporting frequent caffeine consumption. However, so far, caffeine-induced changes in sleepiness, performance, and sleep quality in teenagers have not been systematically investigated.

**Methods:** In a placebo-controlled double-blind within-subject laboratory study, 18 teenagers (15.6 ± 1 years old, self-reported habitual caffeine intake: 241 ± 123 mg/week) ingested 80 mg of caffeine (versus placebo) four hours prior to habitual bedtime after one week of caffeine abstinence. Until bedtime and over 1.5 h after waking up, participants regularly filled in the Karolinska Sleepiness Scale and performed in a psychomotor vigilance and an N-back task (with varying working memory load: 2-back versus 0-back). Subjective sleep quality was measured in the morning by the Leeds Sleep Evaluation Questionnaire. Saliva samples were taken regularly under dim light conditions to determine caffeine and melatonin levels.

**Results:** After intake, caffeine levels increased until bedtime and did not fully return to baseline levels in the morning (*p* < 0.01). Subjective sleepiness was lower, median reaction times were faster, and the number of attentional lapses (RT > 500 ms) reduced (pall < 0.05) after caffeine compared to placebo. In the N-back task, independent of load, a plateau of high performance (correct responses) was reached faster after caffeine compared to placebo (interaction condition * time *p* < 0.05). Finally, the participants indicated a worse quality of falling asleep after caffeine compared to placebo (*p* = 0.05), while no treatment-induced differences were observed in salivary melatonin levels (*p* > 0.1).

**Conclusions:** The data suggest that caffeine intake in the evening (in a dose equivalent to 250 mL of common energy drinks) induces arousal in teenagers, and is mirrored in lower sleepiness and higher attentional performance. While caffeine did not induce a circadian phase delay in the present sample, the worse quality of falling asleep still suggests that a caffeine dose of 80 mg in the evening can be sufficient to contribute to teenagers’ shortened sleep durations, as frequently observed during schooldays.

**Funding:** Swiss National Science Foundation PMPDP1_171364 / 1

### ***11. Changes in Cross-Frequency Coupling Following Closed-Loop Auditory Stimulation during NREM Sleep*** 

KrugliakovaE.^1^VolkC.^1^JaramilloV.^1^SousouriG.^1^HuberR.^1^,^2^1University Children’s Hospital Zürich, 8032 Zürich, Switzerland2Department of Child and Adolescent Psychiatry and Psychotherapy, Psychiatric Hospital, University of Zürich, 8032 Zürich, Switzerland

**Background:** Phase-amplitude coupling between the neocortical slow waves (SW) and faster oscillations is thought to facilitate the coordinated information processing in the multiple brain areas that are necessary for sleep-dependent memory consolidation. Recently, it was demonstrated that by targeting SWs in a particular region with closed-loop auditory stimulation, it is possible to locally manipulate SW activity and interact with ongoing training-induced neuroplastic changes [1]. Capitalizing on this finding, we tested whether closed-loop auditory stimulation might locally affect coupling between SW and power in theta and sigma bands.

**Methods:** For this, we recorded high-density sleep EEG in nine subjects (23 ± 1.3 years old) under two conditions: (1) non-stimulation (SHAM) and (2) closed-loop auditory stimulation (STIM) targeting the up-phase of real-time detected SW in electrode C4 (target electrode). We calculated the modulation index [2], reflecting the association between phases in the 0.75 Hz to 2.75 Hz (SW) range and power in the 4–30 Hz band.

**Results:** In the STIM condition, there was a significant decrease of SW–theta coupling by 31 ± 18% in the frontal channels (*p* = 0.02, *d* = −1.64), which was the area of the strongest default coupling both in STIM and SHAM conditions. In contrast, a significant increase by 124 ± 105% in SW–sigma coupling was observed over the right parietal area, which was located directly posterior to the target electrode (*p* = 0.01, *d* = 1.15).

**Conclusions:** Here, we present initial evidence that closed-loop auditory stimulation locally modulates coupling between SW and sigma in a target region. This opens the way to a variety of future implementations of closed-loop auditory stimulation in different populations with local pathology-related changes in cross-frequency coupling, such as in the case of medial frontal atrophy in older adults [3].

**Funding:** This study was supported by the Swiss Government Excellence Scholarship (Elena Krugliakova), by the Clinical Research Priority Program (CRPP) “Sleep and Health”, the HMZ flagship grant SleepLoop and the SNF Foundation grant 320030_153387 (Reto Huber).

### ***12. Fear Learning, Slow-Wave Sleep, and Trait Anxiety: A Conditioning Study*** 

BirchI. C.^1^LonsdorfT. B.^2^DunsmoorJ. E.^3^ZammitS.^4^,^5^JonesM. W.^6^LewisP. A.^1^1CUBRIC, Cardiff University, CF24 4HQ Cardiff, UK2University Medical Center Hamburg-Eppendorf, 20251 Hamburg, Germany3Department of Psychiatry, University of Texas, Austin, TX 78712, USA4School of Medicine, Cardiff University, CF14 4YS Cardiff, UK5Bristol Medical School, Bristol University, BS8 1TH Bristol, UK6School of Physiology, Pharmacology & Neuroscience, Bristol University, BS8 1TD Bristol, UK

**Background:** Fear conditioning is widely used as a model of fear learning, informing our understanding of post-traumatic stress disorder and other related pathologies. Fear learning has been strongly related to sleep disruption and trait anxiety, however these factors have largely been studied independently. We used a fear conditioning, extinction, and reinstatement design to investigate how sleep and anxiety are associated with fear learning.

**Methods:** Healthy participants (*N* = 20, 18 females, aged 19–28) underwent a two-day fear conditioning paradigm. On day 1, participants were successfully conditioned to fear (CS+) and safe (CS–) stimuli, where CS+ trials were paired with an aversive electric shock (56% reinforcement). Then, overnight sleep at home was monitored with wearable electroencephalography (EEG). On day 2, participants underwent extinction, reinstatement, and re-extinction. Skin conductance response (SCR) was used to measure fear during conditioning and extinction phases.

**Results:** Strength of fear conditioning was associated with subsequent sleep, with better learned CS+/CS– discrimination associated with a lower percentage of slow-wave sleep (SWS%) at a trend level, *p* = 0.054. Overnight consolidation of discrimination was then significantly positively correlated with SWS%, *p* = 0.050. Finally, trait anxiety predicted reinstatement effects. Prospective anxiety was associated with lower CS discrimination after reinstatement, *p* = 0.016, and inhibitory anxiety was associated with a lack of extinction, shown by greater fear responses to both the CS+, *p* = 0.009 and CS-, *p* = 0.007 at the end of re-extinction.

**Conclusions:** Current literature suggests that fear conditioning as a stressful experience impacts subsequent sleep. Here, our findings suggest that the strength of fear learning may affect the proportion of subsequent sleep dedicated to SWS, and in turn, this SWS supports the consolidation of the distinction between safe and fear stimuli. Furthermore, we show that trait anxiety predicts return of fear, and a failure to subsequently extinguish fear responses after reinstatement despite no further fear learning. These data highlight the importance of SWS in fear learning and trait anxiety in return of fear. This could support future work investigating sleep manipulation during SWS to attenuate learned fear.

**Funding:** No funding.

### ***13. Increased Neuronal Signatures of Targeted Memory Reactivation during Slow-Wave Up-States*** 

GöldiM.^1^Van PoppelE.A.M.^1^RaschB.^1^SchreinerT.^2^1Cognitive Biopsychology and Methods, University of Fribourg, 1700 Fribourg, CH2Centre for Human Brain Health, School of Psychology, University of Birmingham, B15 2TT Birmingham, UK

**Background:** It is assumed that the memory function of sleep relies on the spontaneous reactivation of newly acquired memories. Those spontaneous reactivation processes are driven by the cortical slow oscillations, while inducing memory reactivations by re-exposure to learning-associated memory cues (TMR) results in enhanced memory performance. Slow oscillations consist of alternating phases of periods of increased neural firing (up-state) and widespread neural silence (down-state), which roughly corresponds to the positive half wave and negative half wave of the surface slow wave (SW). As the benefits of TMR during sleep might depend on periods of increased neural firing, we hypothesised that cueing foreign vocabularies during SW up-states would lead to enhanced recall performance as compared to words presented during down-states or uncued words, speaking for the crucial role of SW up-states in the reactivation of memories.

**Methods:** Native German speakers learned 120 Dutch–German word pairs in the evening. During subsequent non-rapid eye movement (NREM) sleep, slow waves (SWs) were detected online. One-third of the prior learned Dutch words were repeatedly presented during SW up-states, one-third during down-states, and one-third were not presented at all. Average word length was 500 ms, to fit in either an SW up-state or down-state. After three hours of nighttime sleep, the word knowledge was tested using a cued recall procedure.

**Results:** Words replayed during SW up-states benefited the most as compared to uncued words. Still, down-state replayed words benefited as well, but to a lesser degree than the up-state replayed words. On average, words replayed in an SW up-state were played at 345° of the slow-wave, whereas down-state words were replayed at 130°.

**Conclusions:** Replaying words during the SW up-state phase is related with the strongest memory enhancing effects, suggesting that TMR is more likely to be processed and enhance memory when the stimuli are played during a SW up-state. These findings show that memory enhancement by reactivation during an SW up-state is more stable, but is more variable when the reactivation occurs outside of the up-state. Future analyses will aim for characterising what makes cueing the most beneficial.

### ***14. Daily Caffeine Consumption Reduces Human Grey Matter in Hippocampus Independent of Sleep Pressure*** 

LinY.-S.^1^,^2^,^3^WeibelJ.^1^,^2^LandoltH.-P.^4^,^5^SantiniF.^6^,^7^GarbazzaC.^1^,^2^MeyerM.^1^,^2^,^8^SlawikH.^1^,^2^,^8^BorgwardtS.^3^CajochenC.^1^,^2^ReichertC.^1^,^2^1Centre for Chronobiology, Psychiatric Hospital of the University of Basel, CH-4002 Basel, Switzerland2Transfaculty Research Platform Molecular and Cognitive Neurosciences, University of Basel, CH-4055 Basel, Switzerland3Neuropsychiatry and Brain Imaging, Psychiatric Hospital of the University of Basel, CH-4002 Basel, Switzerland4Institute of Pharmacology and Toxicology, University of Zurich, CH-8057 Zurich, Switzerland5Sleep & Health Zurich, University Center of Competence, University of Zurich, CH-8057 Zurich, Switzerland6Radiological Physics, University Hospital Basel, CH-4031 Basel, Switzerland7Department of Biomedical Engineering, University of Basel, CH-4123 Allschwil, Switzerland8Clinical Sleep Laboratory, Psychiatric Hospital of the University of Basel, CH-4002 Basel, Switzerland

**Background:** Acute caffeine consumption alters sleep–wake regulation by dampening the accumulation of sleep pressure, which is indexed by the reduction in electroencephalographically (EEG)-derived slow wave activity (SWA) during sleep. High sleep pressure has been found to result in reduced grey matter (GM). Therefore, we were interested in whether daily caffeine consumption affects GM, and whether these changes are mediated by sleep pressure.

**Methods:** Twenty male habitual caffeine consumers (aged 26.2 ± 4.1 years, average daily caffeine consumption 471.5 ± 112.24 mg) completed a double-blind randomized study. Each volunteer received both caffeine (3 × 150 mg/day) and placebo for 10 days. After nine days, EEG-derived SWA was measured during nighttime sleep. On day 10, GM was measured via T1-weighted Magnetization-Prepared Rapid Acquisition with Gradient Echo (MPRAGE) in a 3T MRI scanner. The scan took place after 12.5 h of EEG-controlled wakefulness in constant posture and a dim light environment. Differences in total and regional GM between conditions were estimated first. In the next step, we adjusted for SWA (during the first NREM cycle) and cerebral blood flow (CBF) measured by 2D EPI Arterial Spin Labeling (ASL)to test the mediation of sleep pressure on GM and to control for CBF influence on blood-oxygen-dependent-level (BOLD) signals.

**Results:** In the caffeine condition, total GM was lower compared to placebo (*p* = 0.040). Voxel-wise analyses indicated that the most prominent GM decrease in caffeine compared to placebo was in the hippocampus (pFWE = 0.032), along with the left frontal pole, postcentral gyrus, right insula, and the cerebellum at trend (i.e., pFWE < 0.062, pFDR = 0.029.) SWA did not differ between conditions, and correcting for SWA did not affect the coefficient of condition on GM. However, after correcting for total CBF, total GM reduction was no longer significant (*p* = 0.522), while the reduction in hippocampal GM remained significant (pFWE < 0.05) after adjusting for regional CBF.

**Conclusions:** Our data indicate that hippocampal GM reduces in response to daily caffeine consumption. Despite earlier evidence that hippocampal neurogenesis is sensitive to high sleep pressure, the SWA in the previous night’s sleep did not explain the observed hippocampal caffeine-associated GM reduction. It remains to be clarified whether the reduction results from the neuroinflammatory apoptosis or neuroexcitotoxicity via the chronic blockade of adenosine.

**Funding:** Swiss National Science Foundation (320030- 163058).

### ***15. The Effects of Repeated Daytime Caffeine Intake and Its Cessation on Nighttime Sleep*** 

WeibelJ.^1^,^2^LinY.-S.^1^,^2^,^3^LandoltH.-P.^4^,^5^GarbazzaC.^1^,^2^MeyerM.^1^,^2^,^6^SlawikH.^1^,^2^,^6^BorgwardtS.^3^CajochenC.^1^,^2^ReichertC.^1^,^2^1Centre for Chronobiology, Psychiatric Hospital of the University of Basel, 4002 Basel, Switzerland2Transfaculty Research Platform Molecular and Cognitive Neurosciences, University of Basel, 4055 Basel, Switzerland3Neuropsychiatry and Brain Imaging, Psychiatric Hospital of the University of Basel, 4002 Basel, Switzerland4Institute of Pharmacology and Toxicology, University of Zürich, 8057 Zürich, Switzerland5Sleep & Health Zürich, University Center of Competence, University of Zürich, 8057 Zürich, Switzerland6Clinical Sleep Laboratory, Psychiatric Hospital of the University of Basel, 4002 Basel, Switzerland

**Background:** Acute caffeine intake, particularly in the evening, has repeatedly been associated with signs of disturbed sleep such as prolonged sleep latency, reduced sleep duration, decreased EEG delta, and increased sigma power density during non-rapid eye movement (NREM) sleep. However, the effects of repeated caffeine consumption during morning and/or afternoon hours, which is an intake pattern that is prevalent in about 80% of caffeine consumers, remain unknown.

**Methods:** Twenty male healthy regular caffeine consumers (26.4 ± 4.0 years old, habitual daily caffeine intake 474.1 ± 107.5 mg) completed a double-blind, within-subjects study including a caffeine (3 × 150 mg caffeine for nine days), a placebo (3 × placebo for nine days), and a withdrawal condition (3 × 150 mg caffeine for eight days followed by a change to placebo on day nine). After nine days of continuous treatment, EEG-derived sleep structure and intensity were recorded during 8 h of nighttime sleep, beginning 8 h and 15 h after the last caffeine pill in the caffeine and withdrawal condition, respectively. Subjective sleep quality was measured by the Leeds Sleep Evaluation Questionnaire (LSEQ).

**Results:** Sleep latency, total sleep time, and the duration of sleep stages did not significantly differ between conditions (*p* > 0.2). However, the all-night spectral power density in the sigma band (12–16 Hz) during NREM sleep was significantly reduced in both the caffeine and withdrawal condition compared to placebo (*p* < 0.05). Subjective sleep quality did not significantly differ between conditions (*p* > 0.1).

**Conclusions:** Our data indicate that daily caffeine consumption does not lead to clear-cut changes in nighttime sleep structure, when assessed 8 h and 15 h after the last intake. Similarly, subjective sleep quality was not significantly affected by the treatment. However, NREM sleep power density in the sigma range seems to be a sensitive marker mirroring caffeine-induced changes under conditions of daily intake. Its decrease during daily caffeine intake is at first glance in contrast with the increases reported after acute caffeine treatment in earlier studies. However, it can be reconciled within the assumption of a daily overnight withdrawal from the substance.

**Funding:** This work was supported by the Swiss National Science Foundation (320030_163058), the Nikolaus und Bertha Burckhardt-Bürgin-Stiftung, and the Janggen-Pöhn-Stiftung.

### ***16. The Influence of Pre-Sleep Social Media Consumption and Relaxation on Sleep Quality*** 

CombertaldiSelina Ladina^1^OrtAlexander^2^FahrAndreas^2^RaschBjörn^2^1Department of Physiology, University of Fribourg, 1700 Fribourg, Switzerland2Department for Communication and Media Research, University of Fribourg, 1700 Fribourg, Switzerland

**Background:** Due to the current trend to digitalization, it is important to examine effects of the use of respective media channels on different aspects in individuals’ lives. Besides the potential of this trend, digitalization may also have negative effects, as it might reinforce societal gaps through creating a digital divide, foster excessive media use, or decrease trust in media and impair political participation. A recurring question revolves around the negative impact of media use on sleep quality. Sleep is considered to be important for different body functions such as the reconstitution of energy, facilitation of the immune system, and memory consolidation. Intensive media use can lead to a state of cognitive hyperarousal, which has been recently shown for the use of electronic media, smartphones, as well as social media. As cognitive hyperarousal has been reported to negatively affect sleep, we hypothesize that intensive social medial use before sleep will negatively affect objective and subjective sleep quality.

**Methods:** To investigate the influence of social media use on sleep quality, a one-factorial experimental study was conducted. Thirty young and healthy participants spent four nights in a sleep lab. Within experimental conditions, participants either used social media for 30 min before sleep (while wearing blue light-blocking glasses) or followed instructions of a progressive muscle relaxation training for 30 min, or slept directly. In order to get objective sleep parameters, sleep was recorded by polysomnography (EEG, EMG, EOC, ECG, and respiration). Participants rated their subjective sleep quality in the morning.

**Results:** Initial analyses of sleep data show that 30 min of social media use before bedtime impacts sleep architecture: Social media use before sleep reduced the proportion of time participants spent in sleep stage N2, whereas it prolonged the time spent in N1. Further sleep onset latency was reduced after 30 min of relaxation training.

**Conclusions:** Our results sustain the notion that neither relaxation nor social media use immediately before sleep have a strong effect on sleep and sleep-associated functions: sleep latency was reduced after relaxation, whereas N1 was prolonged after social media use. Further analyses will include the comparison between different subgroups of participants in this experiment, e.g., by dividing the group with respect to their intensity of social media use (low versus high).

**Funding:** ERC Starting Grant: MemoSleep 677875.

### ***17. Phase-Coupled Sleep Oscillations within and between Human Hippocampus and Neocortex*** 

CoxRoy^1^RüberTheodor^1^,^2^,^3^StaresinaBernhard P.^4^FellJuergen^1^1Department of Epileptology, University of Bonn, 53127 Bonn, Germany2Epilepsy Center Frankfurt Rhine-Main, Department of Neurology, Goethe University Frankfurt, 60590 Frankfurt am Main, Germany3Center for Personalized Translational Epilepsy Research (CePTER), Goethe University Frankfurt, 60590 Frankfurt am Main, Germany4School of Psychology, University of Birmingham, B15 2TT Birmingham, UK

**Background:** New memories undergo a gradual transfer from the hippocampus (HPC) to the neocortex (NC), with this memory consolidation process preferentially taking place during sleep. Precisely timed neural oscillations interacting within and between these brain structures are thought to mediate this sleep-dependent memory reorganization. However, which components of the large constellation of sleep oscillations instantiate this HPC–NC dialog in the human brain, and via what mechanisms, remains elusive.

**Methods:** We recorded invasive electroencephalography from the HPC and lateral temporal cortex in 10 neurosurgical patients during stages N2 and N3 of non-rapid eye movement sleep, as well as rapid eye movement (REM) sleep.

**Results:** We identified several forms of HPC–NC communication, belonging to two broad classes of interregional oscillatory coordination. First, we observed interregional phase synchrony in the sleep spindle (N3) and theta (N2, REM) frequency bands. Second, we found cross-frequency coupling between the phase of HPC slow oscillations and the amplitude of NC activity spanning the delta, theta, beta, gamma, and ripple ranges (N3).

**Conclusions:** These previously unknown HPC–NC communication lines add to our understanding of the oscillatory organization of the sleeping brain, and may offer a physiological basis for the sleep-dependent reorganization of mnemonic content.

**Funding:** This work was supported by the German Research Foundation (FE366/9-1 to JF) and a Wellcome Trust/Royal Society Sir Henry Dale Fellowship (107672/Z/15/Z to BPS).

## Clinical Science

### ***18. REM-Sleep Related Heart Rate Variability Distinguishes between Insomnia and Normal Sleep*** 

De WitteMarcia^1^Holsboer-TrachslerEdith^2^HatzingerMartin^1^BeckJohannes^2^,^3^PawlowskiMarcel A.^4^MikoteitThorsten^1^,^2^1Psychiatric Services Solothurn and University of Basel, 4503 Solothurn, Switzerland2University of Basel Psychiatric Hospital, Centre for Affective, Stress and Sleep Disorders, 4002 Basel, Switzerland3Clinic Sonnenhalde, 4125 Riehen, Switzerland4Clinic Ingolstadt, Centre of Mental Health, 85049 Ingolstadt, Germany

**Background:** In insomnia disorder, a mismatch between a subjective perception of poor sleep and quite normal objective sleep EEG results is a common finding. The aim of this study was to evaluate the capability of sleep stage-related heart rate variability (HRV), which is a correlate of autonomous nervous system activity, to objectify insomnia.

**Methods:** 47 adults (age: 39.7 ± 12.5 years, 51.1% females) suffering from primary insomnia according to DSM-IV criteria were compared to a control group of 23 adults (age: 37.6 ± 12.3 years, 60.9% females) with no sleep complaints. Sleep was objectively assessed with polysomnography. HRV was assessed in artefact-free five-minute ECG segments of pure sleep stages. Sleep EEG variables and sleep stage-related HRV frequency domain measures, such as the power of high frequency (HF), low frequency (LF), and very low frequency (VLF) bands, were compared between patients and controls.

**Results:** Insomnia and normal sleep did not differ in regard to any of the common measures of objective sleep continuity and sleep architecture, except for a lower amount of REM sleep (%) in insomnia. In the insomnia group, the absolute HRV frequency power over all the frequency bands was suppressed in REM sleep and NREM sleep. Furthermore, in the first available REM sleep ECG segments, the relative HF power was decreased, and the relative VLF power was increased (with large effect sizes, respectively).

**Conclusions:** These results are in line with the theory of hyperarousal in insomnia. Sleep stage-related HRV analysis and specifically HRV in early REM sleep deliver more sensitive biomarkers for insomnia than common sleep EEG variables. Furthermore, REM sleep-related HRV might be a proxy for central autonomous network activity.

**Funding:** No funding.

### ***19. Sleep Determinants of Incident Cardiovascular Events: A Prospective Population-Based Study*** 

HirotsuC.^1^Marques-VidalP.^2^VollenweiderP.^2^WaeberG.^2^BettaM.^3^BernardiG.^3^SiclariF.^1^Haba-RubioJ.^1^HeinzerR.^1^1Center for Investigation and Research in Sleep (CIRS), Lausanne University Hospital (CHUV), 1011 Lausanne, Switzerland2Department of Internal Medicine, Lausanne University Hospital (CHUV), 1011 Lausanne, Switzerland3IMT School for Advanced Studies Lucca, 55100 Lucca, Italy

**Background:** This study aimed to assess the sleep determinants of incident cardiovascular events in a general population sample.

**Methods:** HypnoLaus is a prospective middle-to-older-age population-based cohort in which 2162 participants underwent a complete polysomnography at home, answered sleep questionnaires, as well as had their cardiovascular (CV) profile assessed at baseline and at a five-year follow-up. A local committee adjudicated the development of any CV event (myocardial infarction, acute coronary syndromes, or stroke) following international recommendations. The apnea–hypopnea index (AHI) ≥15 events/h defined obstructive sleep apnea (OSA). A Pittsburgh Sleep Quality Index (PSQI) score >5 defined poor sleep quality. The fast Fourier transformation of non-rapid eye movement (NREM) electroencephalographam (EEG) assessed relative power spectrum components. Autonomic activation during sleep was evaluated through pulse wave amplitude (PWA) drops based on the photoplethysomnographic signal of pulse oximetry. Multivariable-adjusted COX regressions were used for statistical analysis.

**Results:** Of the 1939 participants (56.4 ± 17.7 years, 53.0% women) free of any CV event at baseline, 74 (3.8%) developed a CV event over a five-year follow-up. After adjustment for age, sex, body mass index, systolic blood pressure, smoking, alcohol, metabolic syndrome, dyslipidemia, and hypertension, the following sleep parameters were independently associated with the development of incident CV events: PWA drop index (hazard ratio, HR, for one event/h increase: 0.986 [0.974–0.999], *p* = 0.033), NREM delta EEG power (HR for 1% increase: 0.951 [0.918–0.985], *p* = 0.005), and PSQI>5 (HR vs. PSQI ≤5:2.275 [1.342–3.855], *p* = 0.002). OSA and hypoxia-related sleep parameters were not significantly associated.

**Conclusions:** Impaired vascular reactivity assessed by PWA variations, low NREM delta power, and subjective poor sleep quality are independent predictors of incident CV events in the HypnoLaus cohort.

**Support:** Leenaards fournation, FBM, and SNF.

### ***20. Circadian Modulation of Core Body Temperature in Patients with Central Hypersomnia Disorders*** 

RyserF.^1^,^2^Da Silva AndréG.^1^,^2^MasneufS.^1^BollerV.^1^,^2^MakowskiL.^1^MontvaiE.^1^SchlagerA.-S.^1^WachterA.^1^,^2^GassertR.^2^BaumannC.^1^WerthE.^1^1Department of Neurology, University Hospital Zurich, 8091 Zurich, Switzerland2Department of Health Sciences and Technology, Institute of Robotics and Intelligent Systems, Rehabilitation Engineering Laboratory, ETH Zurich, 8092 Zurich, Switzerland

**Background:** An intact and regular sleep–wake rhythm is the basis for a healthy life. This is ensured by the circadian pacemaker, which drives the sleep–wake cycle to maintain stable vigilance during the day and consolidated sleep during the night. In patients with central hypersomnia, these day–night differences seem less pronounced. However, the knowledge about the interplay between the circadian and the homeostatic sleep–wake regulation in these diseases is limited.

**Methods:** Core body temperature (CBT) has been shown to be a distinct marker of the circadian rhythm. Here, we studied the circadian modulation of CBT over a 24-h day. After a baseline night, a constant condition protocol (controlled room temperature, light <10 lux, low activity (sitting/lying in a bed), isocaloric and temperature-controlled meals) was performed over 40 h. Ten naps of 80-min sleep opportunity were scheduled in equal intervals to control for homeostatic sleep pressure. CBT was continuously measured with a wireless ingestible core body temperature sensor (HQ Inc., Florida). The modulation of CBT was analysed with a cosinor regression (period of 24 h) over the sleep episodes. We present the preliminary results of six unmediated patients with narcolepsy type 1 (Nt1), six patients with idiopathic hypersomnia (IH), and six healthy controls (HC).

**Results:** The results show a clear CBT modulation in all the patient groups. IH showed an increased mean temperature (IH/Nt1/HC: 37.2 °C/37.08 °C/37.08 °C), a higher modulation amplitude (IH/Nt1/HC: 0.24 °C/0.21 °C/0.2 °C) and a shorter acrophase (IH/Nt1/HC: 9.11 h/8.85 h/9.89 h), implying an earlier peak temperature with respect to the subject-specific bedtimes than the control group. No significant group differences were found.

**Conclusions:** This protocol allowed isolating the circadian modulation of core body temperature regulation from external conditions. The results indicate that the circadian rhythm of thermoregulation is maintained even in patients with central hypersomnia disorders. However, these results are based on preliminary data, and other markers than CBT might still reveal a change in circadian pattern.

**Funding:** This research is funded by the swiss national research foundation.

### ***21. Behavioural and Regional EEG Features of Parasomnia Episodes in Disorders of Arousal*** 

CataldiJacinthe^1^LecciSandro^1^BernardiGiulio^1^,^2^Haba-RubioJosé^1^SiclariFrancesca^1^1Center for Investigation and Research on Sleep, Lausanne University Hospital and University of Lausanne, 1011 Lausanne, Switzerland2MoMiLab Unit, IMT School for Advanced Studies Lucca, 55100 Lucca, Italy

**Background and Aims**: Disorders of arousal (DoA) are NREM parasomnias that are characterised by incomplete awakenings from slow-wave sleep (SWS) and abnormal behaviour. Previous work showed that parasomnia episodes (PE) display features of both sleep and wake in different brain areas. However, these observations are based on single episodes, and it is unclear how dissociated patterns of sleep and wakefulness relate to behaviour. Here, we investigated how behaviour and brain activity measured with high-density (hd) EEG differed between PE and normal awakenings (NA) in healthy controls (HC) and DoA patients.

**Methods:** 18 patients with DoA (13F, age 26.4 ± 5.3) underwent a baseline and a second hd-EEG sleep recording coupled with acoustic stimulations after 24 h of total sleep deprivation. Fourteen HC (10F, age 26.5 ± 4.7) underwent a baseline hd EEG recording. Video recordings of all awakenings from SWS were inspected and rated as NA or PE by three independent expert scorers. After a thorough artifact removal procedure, topographical spectral power (2–35 Hz) and behavioural features were compared using paired nonparametric statistics with correction for multiple comparisons and chi square tests, respectively.

**Results:** Compared to awakenings rated as NA (*n* = 47), those rated as PE (*n* = 80) were associated with eye opening, a more rapid onset, a reorienting reaction, perplexity, somniloquia, fear, hallucinations, and environmental interactions (*p* < 0.001 for all, except for fear, *p* < 0.05). Compared to NA in DoA patients, PE were associated with significantly lower sigma (12–16 Hz) and alpha power (8–12 Hz) in posterolateral brain regions (*p* < 0.05, *n* = 11) and marginally increased delta power (2–4 Hz) in central posterior areas. Compared to NA in HC, NA in DoA patients displayed diffusely decreased beta power (18–30 Hz) peaking in frontal brain regions (*p* < 0.05, *n* = 7).

**Conclusions:** Behavioural features that distinguish PE from NA include eye opening, a more rapid onset, a reorienting reaction, perplexity, somniloquia, fear, hallucinations, and interactions with the environment. Spectral analysis suggests that an “incomplete awakening” of posterior brain regions appears to characterise PE. Further analyses will establish how these differential brain activity patterns relate to behaviour and conscious experiences.

**Funding:** SNSF Grant PZ00P3_173955; Théore-Ott Grant.

### ***22. Nocturnal Gamma-Hydroxybutyrate (GHB) Reduces Cortisol Awakening Response and Morning Tryptophan Catabolites in Healthy Volunteers*** 

DornbiererD.A.^1^,^2^,^3^,^5^,^6^BoxlerM.^3^VoegelC.D.^4^StuckyB.^1^,^5^SteuerA.E.^3^BinzT.M.^4^BaumgartnerM.R.^4^BaurD.M.^1^,^5^QuednowB.B.^2^,^6^KraemerT.^3^SeifritzE.^2^,^5^,^6^LandoltH.P.^1^,^5^,^6^†BoschO.G.^2^†1Institute of Pharmacology and Toxicology, University of Zurich, 8057 Zurich, Switzerland2Department of Psychiatry, Psychotherapy and Psychosomatics, Psychiatric Hospital, University of Zurich, 8032 Zurich, Switzerland3Department of Forensic Pharmacology and Toxicology, Zurich Institute of Forensic Medicine, University of Zurich, 8057 Zurich Switzerland4Center for Forensic Hair Analytics, Zurich Institute of Forensic Medicine, University of Zurich, 8057 Zurich, Switzerland5Sleep & Health Zurich, University Center of Competence, University of Zurich, 8091 Zurich, Switzerland6Neuroscience Center Zurich, University Zurich and Swiss Federal Institute of Technology (ETH) Zurich, 8057 Zurich, Switzerland†These authors contributed equally to this work.

**Background:** Gamma-hydroxybutyrate (GHB) is an endogenous GHB-/GABAB receptor agonist that has been approved for the treatment of narcolepsy and proposed for the potential treatment of various neuroimmunological diseases, including Alzheimer’s disease, Parkinson’s disease, fibromyalgia, and depression. Tryptophan catabolites (TRYCATs), the cortisol-awakening response (CAR), and brain-derived neurotrophic factor (BDNF) have all been suggested as peripheral markers of neuroimmunological processes. In addition, GHB was shown to induce a delayed reduction of T helper and natural killer cell counts and alter basal cortisol levels. By contrast, GHB’s effects on TRYCATs, CAR, and BDNF are unknown.

**Methods:** TRYCATs and BDNF plasma levels, CAR, as well as affective state (Positive and Negative Affect Schedule, PANAS) were measured in the morning after a single nocturnal dose of GHB (50 mg/Kg body weight), which was administered at 02:30 to 20 healthy male volunteers. A placebo-controlled, balanced, randomised, double-blind, cross-over design was used.

**Results:** Following GHB, the TRYCATs, indolelactic acid, kynurenine, kynurenic acid, 3-hydroxykynurenine, quinolinic acid, the 3-hydroxykynurenine to kynurenic acid ratio, and CAR were significantly reduced when compared to placebo (*p* < 0.05–0.001, Benjamini–Hochberg corrected). The quinolinic acid to kynurenic acid ratio was reduced by trend. Serotonin, tryptophan, and BDNF levels, as well as PANAS scores, were unchanged after nocturnal GHB challenge.

**Conclusions:** It is concluded that GHB post-acutely affects peripheral markers of neuroimmunological processes. The GHB-induced changes may serve as a model to explain some of GHB’s therapeutic effects in neuropsychiatric disorders involving neuroimmunological pathologies.

**Funding:** No funding.

### ***23. Feeling Awake during Sleep: A High-Density EEG Assessment of Sleep Perception in Good Sleepers and Patients with Paradoxical Insomnia*** 

LecciS.^1^CataldiJ.^1^BernardiG.^2^SiclariF.^1^1Center for Investigation and Research on Sleep, Lausanne University Hospital, 1011 Lausanne, Switzerland2MoMiLab Unit, IMT School for Advanced Studies Lucca, 55100 Lucca, Italy

**Background:** The impression of being awake despite polysomnographically documented sleep is sometimes reported by healthy individuals (good sleepers control, GSC) and to an extreme degree by patients with paradoxical insomnia (ParI). The mechanisms underlying the subjective perception of sleep are not well understood. Here, we asked whether high-density (hd) EEG, a technique with a refined spatial resolution, could identify local patterns of brain activity related to the subjective perception of sleep in ParI and GSC subjects.

**Methods:** Nine ParI (41.54 ± 6.50 (SD)) and 9 GSC (40.61 ± 6.82, age-sex matched, 7F), equipped with hd EEG (256 channels), underwent a baseline and two experimental sleep recordings combined with a serial awakening paradigm. At each awakening, they were asked whether they had felt asleep (FAS) or awake (FAW) immediately prior to the awakening.

**Results:** ParI patients strongly underestimated their total sleep time (*p* = 2.88 × 10^−4^) and overestimated their wake after sleep onset time (*p* = 0.0019). As expected, in experimental nights, FAW instances occurred more often in ParI than in GSC (*p* = 0.0044 for N2 and N3 combined). In both groups, they appear to cluster early in the night (*p* = 0.0138), and tended to be associated with more thought-like and less perceptual conscious experiences compared to FAS (*p* = 0.0642). In GSC, FAWs instances were preceded by short-lasting increases in gamma power (30–45 Hz) in the vertex region, while in ParI patients, FAWs were associated with more diffuse and longer-lasting power increases in a broader frequency range (10 Hz to 45 Hz).

**Conclusions:** We show that the subjective impression of feeling awake during sleep is associated with objective EEG changes (high-frequency power increases), which are rare, short-lasting, and localised in healthy subjects and more frequent, long-lasting and diffuse in patients with paradoxical insomnia. The spatio-spectral characteristics of these changes suggest that feeling awake may result from differential degrees of arousal system activations in the two populations. In light of these results, ParI patients seem to correctly perceive localized “wake-like intrusions”, rather than misperceive their sleep as commonly assumed.

**Funding:** SNSF Grant PZ00P3_173955; Théodore-Ott Grant

### ***24. “Sleep Well, My Dear, to Be Fit for Loving Motherhood”—Insomnia but not Objective Sleep Deficits Correlate with Poor Bonding*** 

MikoteitT.^1^,^2^ClemenJ.^1^BrandS.^2^HösliI.^3^TschudinS.^3^BürkiN.^4^HatzingerM.^1^1Psychiatric Services Solothurn and University of Basel, 4503 Solothurn, Switzerland2University of Basel, University Clinics of Psychiatry, Centre for Affective, Stress- and Sleep Disorders, 4002 Basel, Switzerland3University of Basel, Clinic for Gynecology and Obstetrics, 4506 Basel, Switzerland4Kantonsspital Liestal, Clinic for Gynecology and Obstetrics, 4410 Liestal, Switzerland

**Background:** Insomnia has been acknowledged as a disorder of hyperarousal and impaired REM sleep stability, and there is accumulating evidence that insomnia is associated with disturbed stress coping and emotion regulation. As insomnia is a common complaint in postpartum mothers, the aim of this study was to examine whether subjective or objective maternal sleep quality has an impact on postpartum bonding. Our hypothesis was that insomnia was related to more parenting stress and delayed bonding. We further expected that this was due to a deficit in REM sleep in insomnia.

**Methods:** The sample consisted of 124 healthy mothers (mean age: 32.6 years) at three months after delivery. Subjective insomnia was assessed with the insomnia severity index (ISI), while in a sub-sample of 74 subjects, we also performed in-home sleep EEGs. We further assessed postpartum bonding, parenting stress, and depression.

**Results:** Maternal insomnia was correlated with delayed bonding, specifically increased “anxiety about care”; however, neither insomnia nor anxious bonding were associated with objective measures of sleep. There was a trend for decreased REM sleep in insomnia; however, a decreased amount of REM sleep did not mediate the relation between insomnia and anxious bonding. Moreover, insomnia was significantly related to postpartum depression and parenting stress.

**Conclusions:** In a sample of postpartum mothers, insomnia was related to more perceived stress, depression, and more anxious bonding, which support previous studies that related insomnia to negatively biased emotion processing. Moreover, interventions aiming at calming down hyperarousal in postpartum mothers may have implications for healthy bonding and child development.

**Funding:** Funding by an unrestricted grant from Gottfried and Julia Bangerter-Rhyner Foundation, Berne.

### ***25. The Borderland Between Wakefulness and Sleep*** 

Hertig-GodeschalkAnneke^1^,^2^SkorucakJelena^3^,^4^,^5^MalafeevAlexander^3^,^4^AchermannPeter^3^,^4^,^5^,^6^MathisJohannes^1^†SchreierDavid R.^1^,^2^,^7^†1Department of Neurology, Inselspital, Bern University Hospital, University of Bern, Bern, CH-3010, Switzerland2Graduate School for Health Sciences, University of Bern, Bern, CH-3012, Switzerland3Institute of Pharmacology and Toxicology, University of Zurich, Zurich, CH-8057, Switzerland4Neuroscience Center Zurich, University of Zurich and ETH Zurich, Zurich, CH-8057, Switzerland5Sleep and Health Zurich, University of Zurich, Zurich, CH-8057, Switzerland6The KEY Institute for Brain Mind Research, Department of Psychiatry, Psychotherapy and Psychosomatics, University Hospital of Psychiatry, Zurich, CH-8032, Switzerland7Department of Medicine, Spital STS AG, Thun, CH-3600, Switzerland†Shared last authorship.

Anneke Hertig-Godeschalk

**Background:** The borderland between wakefulness and sleep is poorly defined. Yet, the scoring guidelines of the American Academy of Sleep Medicine (AASM) completely neglect it. We aimed to explore the borderland between wakefulness and sleep by focusing on microsleep episodes (MSEs) visible in the electroencephalogram (EEG) during the maintenance of wakefulness test (MWT). Such MSEs are of potential relevance for diagnosis and could have consequences while driving.

**Methods:** We retrospectively scored MWT trials of 76 randomly selected patients according to the AASM criteria and according to our own scoring criteria for MSEs. The MSE scoring was compared with quantitative EEG analysis.

**Results:** The quantitative EEG analysis enabled a reliable objectification of the visually scored MSEs. The latency to the first MSE was significantly shorter (mean: 15.18 ± 9.44 min) in comparison to the latency of sleep defined according to the AASM criteria (mean: 21.82 ± 11.63 min, *p* < 0.0001, *z* = 5.645). In certain cases, a large difference between the two latencies was observed (ranging from 0.05 to 33.85 min), and a great number of MSEs occurred between the first MSE and sleep (ranging from one to 31). Series of MSEs were more frequent in patients with shorter sleep latencies, while isolated MSEs were more frequent in patients who did not reach sleep.

**Conclusions:** The scoring of MSEs represents a valuable tool in addition to the AASM criteria, enabling an in-depth analysis of the borderland between wakefulness and sleep, which is particularly important in the maintenance of wakefulness test.

**Funding:** This work was supported by the Swiss National Science Foundation (grant 32003B_176323) and the Swiss Innovation Agency/Innosuisse (grant 17864.1 PFLS-LS).

### ***26. Hypoxic Load and Subjective Daytime Sleepiness in the HypnoLaus Cohort*** 

BergerM.^1^HirotsuC.^1^BettaM.^2^BernardiG.^2^SiclariF.^1^Haba-RubioJ.^1^HeinzerR.^1^1Center for Investigation and Research in Sleep (CIRS), Lausanne University Hospital (CHUV), 1011 Lausanne, Switzerland2IMT School for Advanced Studies of Lucca, 55100 Lucca, Italy

**Background:** Although the Epworth Sleepiness Scale (ESS) is widely used to assess excessive daytime sleepiness (EDS) in clinical setting and research studies, its association with subjective and objective sleep parameters is still a matter of debate.

**Methods:** Data from 1982 participants (48.7% men, mean age 58.4 ± 11.1 years) of the population-based HypnoLaus study were analyzed. EDS was defined as an ESS score >10. Ambulatory full polysomnography (PSG) as well as a clinical evaluation including medical history, lifestyle factors, and medication use were performed. Hypoxic load was calculated as the sum of the area under the curve of ≥3% oxygen desaturations/total sleep time. The results were analyzed by multivariate generalised linear models (GLM) to assess means [95% confidence interval] adjusted for age, sex, body mass index (BMI), smoking status, alcohol, coffee consumption, and psychotropic drugs (benzodiazepines, hypnotics, and antidepressants).

**Results:** Compared to participants without EDS (*n* = 1721, 86.8%), participants with EDS (*n* = 261, 13.2%) had higher hypoxic load related to respiratory events during sleep (7.5 [5.5–10.2] versus 6.5 [4.9–8.7] % min/h; *p* < 0.05). A higher hypoxic load in the EDS group was also observed specifically during REM sleep (18.2 [13.2–25.1] versus 14.3 [10.6–19.3] % min/h; *p* = 0.001) as well as a higher percentage of sleep time with an oxygen saturation <90% (T90%: 6.7% [4.5–10.0] versus 5.1% [3.6–7.3]; *p* = 0.01). Other associations with EDS included objective and subjective sleep duration, which were lower in participants with EDS (TST: 402 [393–410] versus 415 [407–423] min; *p* < 0.001) as well as reduced relative delta power in N3 sleep stage (45.4% [43.8–47.1] versus 46.6% [45.1–48.2]; *p* < 0.01). By contrast, the arousal index, apnea–hypopnea index, and oxygen desaturation index did not differ between the two groups.

**Conclusions:** Hypoxic parameters such as hypoxic load and T90% are associated with higher EDS in our large middle-to-older age population-based sample.

**Support:** Leenaards Foundation, FBM, and SNF.

### ***27. Epic Dreaming Disorder: Clinical, Polysomnographic and Dream Features in 12 Patients*** 

KrämerC.CataldiJ.LecciS.SiclariF.Centre of Investigation and Research on Sleep, Lausanne University Hospital and University of Lausanne, 1011 Lausanne, Switzerland

**Background:** Epic dreaming disorder (EDD) is a condition in which patients complain of excessive and relentless dreaming throughout the night that leaves them feeling tired the next day. Although regularly encountered in clinical practice, it is poorly documented, and no systematic assessment of dreaming has been performed in this population so far.

**Methods:** Here, we investigated the clinical characteristics of 12 EDD patients and 12 healthy gender-matched and age-matched controls (HC) through medical interviews and questionnaires. Seven EDD patients and 12 HC also underwent a baseline high-density EEG sleep recording, and six ED and eight HC completed one to two additional recordings combined with a serial awakening paradigm to inquire about dream experiences.

**Results:** EDD patients were predominantly middle-aged females (81.81%, mean age ± SD: 35.36 ± 8.32). The disorder was lifelong in half of the patients and chronic in the remainder (mean duration 16.4 ± 8.44 years). EDD patients scored higher on anxiety and depression scales, and complained of lower sleep quality, as well as higher daytime sleepiness and fatigue. Sleep recordings revealed a higher microarousal index in EDD patients compared to HC (22.74/h ± 11.9 versus 12.71/h ± 5.11, *p* = 0.024), but an otherwise preserved sleep architecture. The proportion of awakenings yielding dream reports was similar to control subjects (74.38 ± 21.91% versus 89.25 ± 13.76%, *p* = 0.106), but EDD patients presented significantly fewer dreams without recall of content in stage N2 (23.7 ± 29.11% versus 56.73 ± 18.52%, *p* = 0.023) and a trend toward a higher proportion of dreams with recalled content in rapid eye movement (REM) sleep (92.06 ± 13.69% versus 59.22 ± 40.81%, *p* = 0.085). Dream reports of EDD patients contained significantly more words overall and in REM sleep (overall: 49.57 ± 32.58 versus 17.29 ± 8.78, *p* = 0.014, REM: 74.04 ± 44.27 versus 23.38 ± 8.8, *p* = 0.02), and were rated as richer, more thought-like, and longer.

**Conclusions:** Our results do not sustain the hypothesis that EDD patients dream more than controls. Rather, they appear to recall the content of their dreams better in both REM and non-REM (NREM) sleep, and to display qualitative differences in dream characteristics. The increased microarousal index may explain why EDD subjects recall a higher number of dreams compared to control subjects.
